# Mitochondrial Donation within a Reproductive Care Pathway for mtDNA Disease

**DOI:** 10.1056/NEJMoa2503658

**Published:** 2025-07-16

**Authors:** Robert McFarland, Louise Hyslop, Catherine Feeney, Rekha N Pillai, Emma L Blakely, Eilis Moody, Matthew Prior, Anita Devlin, Robert W Taylor, Mary Herbert, Meenakshi Choudhary, Jane A Stewart, Douglass M Turnbull

**Affiliations:** Mitochondrial Research Group, Translational and Clinical Research Institute, Faculty of Medical Sciences, Newcastle University, Newcastle upon Tyne, UK; NHS Highly Specialised Services for Rare Mitochondrial Disorders, Newcastle upon Tyne Hospitals NHS Foundation Trust, Newcastle upon Tyne, UK; Newcastle Fertility Centre, Newcastle upon Tyne Hospitals NHS Foundation Trust; International Centre for Life, Newcastle, UK; NHS Highly Specialised Services for Rare Mitochondrial Disorders, Newcastle upon Tyne Hospitals NHS Foundation Trust, Newcastle upon Tyne, UK; Newcastle Fertility Centre, Newcastle upon Tyne Hospitals NHS Foundation Trust; International Centre for Life, Newcastle, UK; NHS Highly Specialised Services for Rare Mitochondrial Disorders, Newcastle upon Tyne Hospitals NHS Foundation Trust, Newcastle upon Tyne, UK; Newcastle Fertility Centre, Newcastle upon Tyne Hospitals NHS Foundation Trust; International Centre for Life, Newcastle, UK; Newcastle Fertility Centre, Newcastle upon Tyne Hospitals NHS Foundation Trust; International Centre for Life, Newcastle, UK; Great North Children's Hospital, Newcastle upon Tyne Hospitals NHS Foundation Trust, Newcastle upon Tyne, UK and Translational and Clinical Research Institute, Faculty of Medical Sciences, Newcastle University, Newcastle upon Tyne, UK; Mitochondrial Research Group, Translational and Clinical Research Institute, Faculty of Medical Sciences, Newcastle University, Newcastle upon Tyne, UK; NHS Highly Specialised Services for Rare Mitochondrial Disorders, Newcastle upon Tyne Hospitals NHS Foundation Trust, Newcastle upon Tyne, UK; Newcastle Fertility Centre, Newcastle upon Tyne Hospitals NHS Foundation Trust; International Centre for Life, Newcastle, UK; Biosciences Institute, Newcastle University, Biomedicine West Wing, Centre for Life, Times Square, Newcastle upon Tyne, UK; Department of Anatomy and Developmental Biology, Monash Biomedicine Discovery Institute, Monash University, Melbourne, VIC, Australia; Newcastle Fertility Centre, Newcastle upon Tyne Hospitals NHS Foundation Trust; International Centre for Life, Newcastle, UK; Newcastle Fertility Centre, Newcastle upon Tyne Hospitals NHS Foundation Trust; International Centre for Life, Newcastle, UK; Mitochondrial Research Group, Translational and Clinical Research Institute, Faculty of Medical Sciences, Newcastle University, Newcastle upon Tyne, UK

## Abstract

Pathogenic mitochondrial DNA (mtDNA) variants are a common cause of severe, often fatal, inherited metabolic disease. A reproductive care pathway was implemented to provide women carrying pathogenic mtDNA variants with reproductive options. Twenty-five women with pathogenic mtDNA variants are in process or have completed pronuclear transfer (and thus receipt of a mitochondrial donation), with 8 live births. All 8 children were healthy at birth, with no or low levels of mtDNA heteroplasmy in blood. One child developed hyperlipidemia (also present in mother during pregnancy) and cardiac arrhythmia; both conditions responded to treatment. Another developed infant myoclonic epilepsy with spontaneous remission. The oldest child is 5 years and all have made normal developmental progress.

Pathogenic mitochondrial DNA (mtDNA) variants are a common cause of severe inherited metabolic disease.^1,2^ Mitochondrial DNA is present in multiple copies within each cell and, for many pathogenic variants, the relative abundance (heteroplasmy) of mutated copies is a key factor in determining the severity of disease; with higher levels of heteroplasmy causing disease in a diverse range of organs.^3^ However, other pathogenic mtDNA variants are typically homoplasmic (all copies are mutated) and exhibit a characteristic pattern of isolated organ involvement with variable penetrance.^4,5^

Despite the prevalence and severity of mtDNA disease, there are limited treatments.^6^ The exclusive maternal transmission of mtDNA means that children of women with homoplasmic pathogenic mtDNA variants are obligate homoplasmic carriers. The outcome for children born to mothers with heteroplasmy is less predictable; during female germ cell development, a mitochondrial genetic bottleneck leads to a random shift in heteroplasmy, resulting in offspring with variable mutant loads.^7,8^

For women considering treatment through assisted reproductive technology to lower the risk of serious mtDNA-related disease in their offspring, options include preimplantation genetic testing (PGT).^9,10^ However, for women with high mtDNA heteroplasmy or homoplasmic pathogenic mtDNA variants, PGT is not an option and mitochondrial donation has been considered as a possibility.^11^

After careful preclinical testing of mitochondrial donation using pronuclear transfer^12^ and a change in the Human Fertilisation and Embryology Act (UK), a new comprehensive National Health Service (NHS) care pathway^13^ was established to provide informed reproductive choices for women; one of these choices is to accept a mitochondrial donation through pronuclear transfer. Here, we describe this pathway and some data: ie, the genetic and clinical outcomes of pregnancies and children born following mitochondrial donation. In an accompanying article in this issue of the *Journal*, Hyslop et al.^14^describe the corresponding embryology data.

## Methods

The mitochondrial reproductive care pathway implemented in 2017 (the NHS Highly Specialised Mitochondrial Reproductive Care Pathway) is available to all women living in the UK who harbor pathogenic mtDNA variants, with an underlying principle to provide these women with informed reproductive choice that can be implemented in a regulated environment.^13,15^ The pathway comprises the Mitochondrial Reproductive Advice Clinic (MRA-C) and the Mitochondrial Assisted Reproductive Technology Clinic (MART-C) where women and their partners undergo detailed clinical review (Supplementary Methods).

As part of the licencing conditions, women proceeding with mitochondrial donation are offered close monitoring of the pregnancy and follow-up of the offspring (Supplementary Methods; Supplementary Figure). Throughout the process, any health concerns are discussed and, when indicated, assessed by the NHS Highly Specialised Mitochondrial Reproductive Care Pathway team including assessment of the long-term consequences for all children born.

## Results

### Reproductive Care Pathway

**Figure 1:**
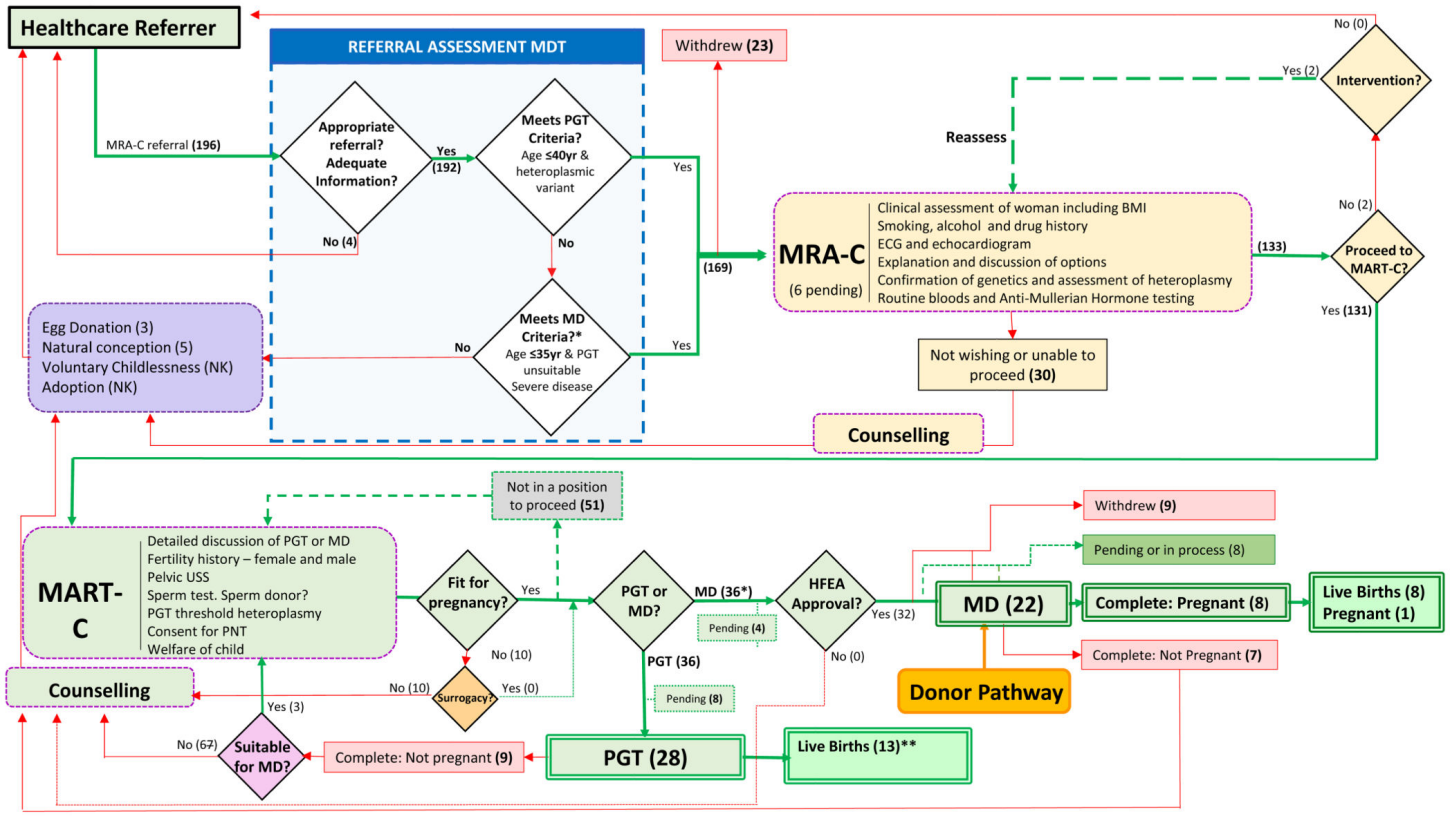
Reproductive care pathway for women with pathogenic mtDNA variants. Women are referred for reproductive advice and potential ART to limit the transmission of the pathogenic mtDNA variant. The numbers referred and progressing through each part of this pathway are shown. Women are assessed at the MRA-C or MART-C clinics for assessment prior to either PGT or PNT. *Includes three women who had PGT but had no embryos below threshold and were subsequently referred for mitochondrial donation. **Births and pregnancies since start of Mitochondrial Reproductive Care Pathway. Completed means those women who have failed to become pregnant despite a full course of treatment. 51 women who were not able to proceed with either PGT or mitochondrial donation, within their reproductive capacity, can be referred back to MRA-C. MD – mitochondrial donation.

196 referrals, broadly reflecting the social and ethnic demographic of the UK population (Supplementary Table S1), were reviewed by the Referral Assessment Multidisciplinary Team (MDT), 4 of which were not considered further due to insufficient referral information or an incorrect diagnosis (Figure 1). Prior to the offer of an appointment in the MRA-C, 23 women withdrew from the process and 9 had become pregnant. Six women have appointments pending.

Of the 163 women evaluated in the MRA-C, 133 women have received or are receiving care in the MART-C, two of whom are participating in healthy-lifestyle programs to decrease their body mass index (Figure 1). Thirty were unable or opted not to proceed further; most of these women viewed the process as “fact finding” to inform their future reproductive decision-making, and three had conceived naturally prior to their planned consultation in MART-C.

Following MART-C assessment of their reproductive options, 51 women did not proceed in the program. Reasons included limitations to performing IVF such as a very low ovarian reserve (n=6), poor maternal health (n=7) and age (n=1). Many women declined at this stage due to personal priorities and timing, often with a desire to proceed in the future. Five women were offered a reproductive intervention but declined without providing a reason.

Of the 70 women proceeding with a reproductive option, 36, in whom 10 different pathogenic mtDNA variants were represented, were offered PGT, resulting in the birth of 13 children (Figure 1) since the start of the care pathway.

### Mitochondrial Donation

#### Applications for Mitochondrial Donation

All 32 applications submitted to the HFEA for mitochondrial donation using pronuclear transfer were approved; four applications are pending (Figure 1). Ten pathogenic mtDNA variants were represented across the 32 women: (NC_012920.1): m.11778G>A (n=9); m.3243A>G (n=7); m.3460G>A (n=4); m.8344A>G (n=4); m.4300A>G (n=3); m.3250T>C (n=1); m.3260A>G (n=1); m.3635G>A (n=1); m.7510T>C (n=1); and m.14484T>C (n=1). Of these 32 women, three had previously had embryos genetically tested (one of them, before the introduction of care pathway) but did not have any transferred due to high levels of heteroplasmy in each tested embryo.

#### Pregnancies After Mitochondrial Donation

Ultrasound scan at 7 weeks confirmed 8 pregnancies, from which there have been 8 live births (4 female and 4 male), including a set of monozygotic twins, and 1 ongoing pregnancy (Table 1). There have been no miscarriages following ultrasound confirmation of pregnancy at 7 weeks’ gestation. Six pregnancies were without complication. One woman, homoplasmic for m.4300A>G, had a pre-existing hypertrophic cardiomyopathy and experienced paroxysmal atrial fibrillation during preparation for embryo transfer. This required treatment with a beta blocker and thromboprophylaxis. This patient also experienced a serious complication of pregnancy: extreme hypertriglyceridemia (serum triglyceride 77.17 mmol/L (NR 0.55-1.9), serum cholesterol 23.2 mmol/L (NR <5 mmol/L)), which improved with restriction of dietary fat to less than 20g/day and resolved within a week postpartum.

#### Follow-up of Children

##### Assessment of mtDNA variant levels

All babies were born between 36+1 and 42+2 weeks by normal vaginal delivery or elective cesarean section for potential maternal-health concerns or, in one case, placenta previa.

Newborn and placental weights and Apgar scores were within normal ranges (Table 2). The level of heteroplasmy, available in each of 8 children, was below the threshold for clinical disease. Five children had undetectable levels at birth. A child whose mother was homoplasmic for the m.4300A>G variant had levels of 5% and 9% in blood and urine, respectively, at birth, and undetectable levels in blood at 18 months. A child (with m.3260A>G) had 16% and 20% heteroplasmy in blood and urine respectively, at birth, and another child (m.11778G>A) had 12% and 13% heteroplasmy in blood and urine respectively, at birth.

##### Follow-up of Children

All 8 neonates were healthy at birth and appear to be making normal developmental progress (Supplementary Results). To date, 5 children have had no reported medical problems. An infant born to a mother with the m.3460G>A variant, who had no detectable maternal mtDNA at birth, experienced brief startles (head and neck flexion with eye blink) from age 7 months. Neurologic, developmental and general examination were normal, as were routine blood tests and interictal electroencephalogram. The child was diagnosed with myoclonic epilepsy of infancy, a rare, typically self-limiting condition, which occurs in otherwise healthy and developmentally normal children.^15^ After 3 months, myoclonic jerks ceased without treatment, and developmental progress remains normal in all domains. Another child had a urinary tract infection that responded promptly to antibiotic treatment.

One breastfed infant, born to the woman with m.4300A>G variant, who experienced gestational severe hypertriglyceridemia, had prolonged jaundice with mildly elevated levels of liver enzymes (elevated alanine transaminase and gamma-glutamyl transferase with conjugated hyperbilirubinemia), an enlarged hyperechogenic liver (indicative of hepatic steatosis) on ultrasound scan and hyperlipidaemia (serum triglyceride 7.5 mmol/L; normal < 1.15 mmol/L). Dietary restriction of fat intake led to resolution of the hyperlipidaemia and fatty liver over the course of 3 months. An echocardiogram, performed at approximately 4 months old, revealed a dilated left ventricle. This prompted a detailed pediatric cardiology assessment that demonstrated ventricular pre-excitation (Wolff Parkinson White pattern), and atrial tachycardia with secondary left ventricular dilatation, although the patient was asymptomatic. Extensive genetic investigations, including genome sequencing, did not identify a hereditary cause for this cardiac phenotype. Two independent pediatric cardiologists concluded that the atrial tachyarrhythmia was responsible for the enlarged left ventricle with reduced contractility. Treatment with anti-arrhythmic medications has resulted in the left ventricle returning to normal morphology and function. This child was assessed at an adjusted age of 18 months 12 days, using the Bayley-III assessment of infant and toddler development (3^rd^ edition):^17^ the scores were within the normal range. Both hearing and vision were assessed as normal by bedside testing. This child remains well and continues to develop normally. Anti-arrhythmic treatment is being slowly weaned with a view to possible ablative therapy in the future. One other child has had an 18-month neurodevelopmental assessment that was normal in all domains. Planned cardiology assessments have been normal.

## Discussion

Women at risk of transmitting severe mtDNA disease to their children should have the opportunity to make informed choices about their reproductive options: prenatal testing, pre-implantation genetic testing, mitochondrial donation, egg donation, adoption and deciding not to have children. Multidisciplinary advice regarding the optimal choice for an individual woman is tailored to the specific mtDNA variant and the woman’s own views on risk reduction. In this study, many of the women who were eligible for mitochondrial donation or PGT decided, after counseling, not to proceed in the pathway. Because mitochondrial donation and PGT involve in vitro fertilization, the success of which is inversely correlated with age,^18,19^ we would encourage this type of early fact-finding discussion. Whilst this study involved women from the UK, we believe the results are generalizable to other countries.

There is a theoretical risk of deterioration in health during pregnancy for women with pathogenic mtDNA variants, with a higher risk of pregnancy complications and early delivery^20^. In the 7 pregnancies, there have been no reported complications, although one patient with hypertrophic cardiomyopathy developed intermittent atrial fibrillation. This patient also developed a severe complication of extreme hyperlipidemia during pregnancy.^21^ Despite extensive metabolic and genetic investigations (Supplementary Results), the cause of this hyperlipidaemia has not been identified. We are not aware of reports of extreme hyperlipidaemia associated with this or any other mtDNA variant during pregnancy.

The 8 infants were healthy at birth and are developing normally (Supplementary Results). Our data support a marked reduction in the transmission of pathogenic mtDNA variants after mitochondrial donation, with heteroplasmy either undetectable or well below a threshold likely to cause disease. We were only able to sample easily accessible tissues and do not know whether these tissues are entirely representative.

One child born to a mother with m.4300A>G related disease had hyperlipidaemia (also noted in her mother during pregnancy) and cardiac arrhythmia; both conditions have been successfully treated. Primary cardiac arrhythmia is not a known feature of the m.4300A>G variant (rather, patients with this variant are at risk of progressive hypertrophic cardiomyopathy).^5^ None of the other children demonstrated cardiac problems. We have instigated a pediatric cardiology review at 3-6 months in all children born following mitochondrial donation. Another child developed myoclonic epilepsy of infancy, a self-limiting condition which has resolved.

Although there are concerns about the safety of mitochondrial donation, including nuclear-mitochondrial mismatch and reversion to the original maternal mitochondrial genotype^22,23^, it is challenging to establish cause and effect of adverse health outcomes in babies born. First, the procedures of conventional assisted reproductive technology are associated with an increased incidence of congenital anomalies, most notably of the cardiovascular system.^24^ Second, some pathogenic mtDNA variants are associated with an increased incidence of pregnancy complications which can cause adverse child health outcomes.^20^ Third, the risk of cardiovascular defects is increased by exposure during early pregnancy to maternal metabolic conditions such as diabetes^25^ and hyperlipidaemia.^26^ It is of interest that one child born after PGT, where the pregnancy was complicated by diabetes mellitus, had a congenital cardiac defect.

In conclusion, pronuclear transfer, a form of mitochondrial donation, is effective in reducing the level of pathogenic mtDNA variant to substantially below the threshold for clinical disease in the offspring of women with homoplasmic (or high heteroplasmic) levels. We are assessing, over the long term, the health and extent of heteroplasmy (if detectable) of the offspring. Indeed, the role of mitochondrial donation as a choice for women with a heritable pathogenic mtDNA variant will only be established with the availability of additional data.

Disclosure forms provided by the authors are available with the full text of this article at NEJM.org.

## Supplementary Material

Supplement

## Figures and Tables

**Table 1:** Characteristics of Women With Pregnancies after Pronuclear Transfer

mtDNA Variant (NC_012920.1)	Heteroplasmy (%)				Age (Yr)	Disease in Family	Outcome MD
	Blood	Urine	Buccal	Muscle			
m.3260A>G	36	79	N/A	94	27	Severe cardiomyopathy	Live birth
m.3460G>A	67	89	86	N/A	31	Blindness	Live birth
m.4300A>G	Hom	Hom	Hom	N/A	36	Fatal cardiomyopathy / CD	Live birth
m.4300A>G	Hom	Hom	N/A	N/A	34	Fatal cardiomyopathy / CD	Live birth
m.11778G>A	74	82	N/A	81	35	Blindness	Live birth
m.11778G>A	Hom	Hom	Hom	N/A	30	Blindness	Live births (twins)
m.11778G>A	94	Hom	Hom	N/A	31	Blindness	Live birth
m.11778G>A	Hom	Hom	Hom	N/A	33	Blindness	Pregnant

Key: N/A = not assessed; CD = child death; Hom = homoplasmic within limits of detection >97%

**Table 2:** Details of Pregnancy and Births of Infants after Pronuclear Transfer

mtDNA variant (NC_012920.1)	Max. Mat Het (%)	Sex	Antenatal Ultrasound	Gestation (wk+d)	Mode of Delivery	Birth Wt (g)	Apgars			Heteroplasmy (%)	
							1m	5m	10m	Blood	Urine
m.3260A>G	94	M	Normal	38	EL LSCS	3220	-	9	10	16	20
m.3460G>A	89	F	Normal	38+6	NVD	3090	-	9	10	ND	ND
m.4300A>G	>97	F	Abnormal*	38	EL LSCS	2815	10	10	10	5	9
m.4300A>G	>97	M	Normal	38+6	EL LSCS	3210	9	10	10	ND	ND
m.11778G>A	82	F	Normal	42+2	EL LSCS	3665	5	7	9	ND	ND
m.11778G>A	>97%	M	Normal	36+1	EL LSCS	2460	5	9	10	ND	NA
		M	Normal	36+1	EL LSCS	2190	5	9	10	ND	NA
m.11778G>A	>97%	F	Normal	36+2	NVD	3330	5	9	9	12	13

* suspected renal tract abnormality that was not detectable on post-partum scan

Max.Mat Het = maximum maternal variant heteroplasmy detected in any sample 1m, 5m and 10m = One, five and ten minutes respectively ND = Not detected NA = Not available >97 = Homoplasmic within the limits of detection
