# Supplementary material for: Mitochondrial Donation within a Reproductive Care Pathway for mtDNA Disease
Source: N Engl J Med. Author manuscript; Available in PMC 2025 Jul 31. (PMC7617939; doi:10.1056/NEJMoa2503658)
Supplement: Supplement [file EMS206184-supplement-Supplement.pdf]

SUPPLEMENTARY APPENDIX

CONTENTS

List of investigators……………………………………………………………………………………………..2

Supplementary methods

Mitochondrial Reproductive Advice Clinic (MRA-C) and Mitochondrial Assisted Reproductive Technology clinic (MART-C)…………………………………………3

Regulation of mitochondrial donation…………………………………………………………..4

Egg donors for mitochondrial donation…………………………………………………………..5

Monitoring of pregnancy and follow up of any children born…………………………..5

Quantification of mtDNA heteroplasmy…………………………………………………………..6

Supplementary Results

Genetic tests for hyperlipidemia……………………………………………………………………6

Developmental progress……………………………………………………………………………….7

Supplementary figure……………………………………………………………………………………………..8

A. Pregnancy pathway for patients undergoing Mitochondrial Donation

B. Pathway for follow up of child born after Mitochondrial Donation

Supplementary table……………………………………………………………………………………………9

References……….10

List of investigators

Robert McFarland Ph.D., Louise Hyslop Ph.D., Catherine Feeney MSc, Rekha N Pillai Ph.D., Emma L Blakely Ph.D., Eilis Moody MSc., Matthew Prior Ph.D., Anita Devlin M.D., Robert W Taylor D.Sc., Mary Herbert Ph.D., Meenakshi Choudhary Ph.D., Jane A Stewart M.D., Douglass M Turnbull Ph.D.

Mitochondrial Research Group, Translational and Clinical Research Institute, Faculty of Medical Sciences, Newcastle University, Newcastle upon Tyne, UK (RM, RWT, DMT), the NHS Highly Specialised Services for Rare Mitochondrial Disorders, Newcastle upon Tyne Hospitals NHS Foundation Trust, Newcastle upon Tyne, UK (RM, CF, ELB, RWT), the Newcastle Fertility Centre, Newcastle upon Tyne Hospitals NHS Foundation Trust; International Centre for Life, Newcastle, UK (LH, RP, EM, MP, MH, MC, JS), the Great North Children's Hospital, Newcastle upon Tyne Hospitals NHS Foundation Trust, Newcastle upon Tyne, UK and Translational and Clinical Research Institute, Faculty of Medical Sciences, Newcastle University, Newcastle upon Tyne, UK (AD), the Biosciences Institute, Newcastle University, Biomedicine West Wing, Centre for Life, Times Square, Newcastle upon Tyne, UK (MH), and the Department of Anatomy and Developmental Biology, Monash Biomedicine Discovery Institute, Monash University, Melbourne, VIC, Australia (MH).

Methods

Mitochondrial Reproductive Advice Clinic (MRA-C) and Mitochondrial Assisted Reproductive Technology clinic (MART-C)

Entry point to the service is via clinical referral of women with pathogenic mtDNA variants and each referral is assessed by a multidisciplinary team comprising fertility and mitochondrial clinicians. Appointments at both the Mitochondrial Reproductive Advice Clinic (MRA-C) and Mitochondrial Assisted Reproductive Technology Clinic (MART-C) are arranged on the same day, to reduce travel burden, along with the necessary investigations. At MRA-C, women and their partners are provided with verbal, written information on the options available to them including egg donation, natural conception, decision not to have children, adoption, PGT and mitochondrial donation. Current health status is assessed at this clinic with specific regard to the physiological implications of pregnancy on health. Women are informed of the possible complications of pregnancy where these have been established e.g. m.3243A>G carriers.[1] Further clinical investigations (e.g. bloods, ECG, echocardiogram) and confirmation of mtDNA variant heteroplasmy, in three different tissues (blood, urine and buccal), are also requested from this clinic. This is followed by a multidisciplinary team meeting before the MART-C to ensure effective communication and consideration of issues such as counselling, patient safeguarding and welfare of the child. MART-C assesses in detail the reproductive health of both partners including estimation of ovarian reserve (through blood Anti-Mullerian Hormone (AMH) levels and a transvaginal pelvic ultrasound scan (for antral follicle count) and a sperm assessment. In keeping with HFEA guidance, an expert multidisciplinary team, including mitochondrial and fertility specialists, provide a detailed explanation of PGT and mitochondrial donation as potential reproductive options. The discussion expands on recommended safe levels of heteroplasmy in blastomeres for PGT (mtDNA variant dependent[2]), mitochondrial donation as a novel technique with limited outcome data[3], the risk of mtDNA reversion to the maternal mitochondrial genotype[4,5] and follow-up of children born following mitochondrial donation. Within the context of the personal and family experience of disease and the results of fertility and mtDNA heteroplasmy investigations, the woman and her partner will come to a decision with the clinical team regarding the most appropriate treatment option for them. If appropriate, women are offered donor sperm.

In England, NHSE funding for PGT is offered where relevant when the female partner is ≤40 years when treatment begins. This is based on the known risks of decline in oocyte yield and higher rates of aneuploidy with increasing age. In a new intervention these impacts can be critical. Although at the outset similar clinical criteria were applied to women having mitochondrial donation, more recently a pragmatic age limit of ≤35 years has been applied to account for the prolonged process.[6,7]

The couples are followed up in the MART-C after a period of several weeks during which they can reflect on the information from the previous appointment and to formulate additional questions. If they wish to proceed with assisted reproductive technology (ART) then a treatment plan is mapped out based on the intended intervention (PGT or mitochondrial donation). When necessary, advice is sought from relevant clinical specialties such as a medical obstetrics, anaesthetics, neurology or cardiology, so that ART can be performed safely, and pregnancy managed appropriately. The couple may have to attend a pre-pregnancy clinic to optimise existing clinical conditions prior to ART being performed. At this point, if the woman's clinical condition means that it is not safe to carry a pregnancy, then consideration of surrogacy may be discussed with the couple.

The clinics are set up to provide support for the couple throughout the treatment process including one routine session with the fertility counsellor for patients undergoing mitochondrial donation followed by access to the service on an 'as and when required basis. There is round the clock access for advice and support with a dedicated nursing mailbox as a point of contact for the couple.

Regulation of Mitochondrial Donation

Human Fertilisation and Embryology (Mitochondrial Donation) Regulations 2015 came into force on 29th October 2015 after both Houses of the UK Parliament approved the legislation. Additional assessment by an expert panel convened by the HFEA was required before the clinical service was established in 2017. Mitochondrial donation can only proceed if an application for each patient is approved by the HFEA Statutory Approvals Committee[8]. Applications are assessed based on whether the mitochondrial variant is pathogenic, whether it is homoplasmic or very high heteroplasmy such that PGT is inappropriate, and the child is at risk of disease serious enough to warrant mitochondrial donation. While for the more common pathogenic mtDNA variants this is relatively straightforward, additional

meta-analysis of publicly available and unpublished data has been required in cases of unusual mtDNA variants. Each application is considered by an independent peer reviewer who is a mitochondrial expert, and by the committee itself (which includes senior clinicians with experience of looking after patients with genetic disease). The committee also receives input from an independent scientific advisor.

Egg donors for Mitochondrial Donation

The egg donors were either recruited anonymously via the Newcastle Fertility Centre egg donation programme or were known to the patient. In addition to checking ovarian reserve and for infectious diseases, mtDNA sequencing was performed in two samples (blood and urine) to test for pathogenic mtDNA variants. Full mtDNA next generation sequencing was carried out in the UKAS-accredited NHS Highly Specialised Mitochondrial Diagnostic laboratory, as set out in the NHS England National Genomic Test Directory[9]. mtDNA variant classification is according to ACMG/AMP and the recently updated UK ACGS best practice guidelines[10], aligning with specific guidelines for the molecular genetic diagnosis of mitochondrial disease[11]. None of the donors who donated their eggs had pathogenic mtDNA variants, likely pathogenic mtDNA variants or mtDNA variants of uncertain significance. All donors were counselled in advance of genetic testing with regards to these possible outcomes and follow up counselling was also provided.

Monitoring of pregnancy and follow up of any children born

Clinical oversight for women undergoing mitochondrial donation commences from approximately 7 weeks gestation when a viable pregnancy is first confirmed by ultrasound scan and includes, routine ultrasound scans throughout pregnancy, regular contact throughout pregnancy, details of delivery, birth weight and routine health and developmental assessments in infancy (**Figure 1A**). Women are offered prenatal diagnosis to assess genotype after mitochondrial donation, but no woman has wished to proceed with this testing. At birth, at 18 months and 5 years blood and urine samples are taken for assessment of mtDNA genotype.

In UK, shortly before or after birth, the family are given a personal child health record known as the "red book". The child's weight and height, vaccinations are recorded but there is also a developmental milestones section that allows parents to observe and assess if their

child's development is following the expected path. Newborn hearing tests are routinely conducted.

At 6-8 weeks a thorough physical examination is conducted by the primary care physician and at 9-12 months and 2-2.5 years developmental assessments are conducted by Health Visitors (HV); usually specially trained midwives or nurses. In addition, there are vaccination visits at 8 weeks, 12 weeks, 16 weeks and 12 months old, and before starting school.

The specific additional follow up of children born following mitochondrial donation was based on multiple discussions with families affected by mtDNA related disease and the regulator (HFEA) (**Figure 1B**). Assessment at 18 months was not designed to detect mitochondrial disease, but to ascertain if mitochondrial donation is associated with any effects on early development. To detect the protean clinical manifestations of a wide range of mtDNA variants with variable age of onset would be impossible without over-medicalising the child and providing intensive follow-up for many years. The compromise is to have vigilant oversight of routine health encounters between the mother/child and healthcare professionals as described above, together with three formal assessments: a review by a paediatric cardiologist at 3-6 months; and a detailed neurodevelopmental assessment by two paediatric neurologists using the Bayley Scales of Infant and Toddler Development 3rd Edition (Bayley-III) at 18 months[12] and a further formal developmental assessment at 5 years.

In the UK, PGT is an established accepted ART procedure, and it is parental choice in relation to follow up and measurement of heteroplasmy in the child's tissues.

Quantification of MtDNA heteroplasmy

Quantitative pyrosequencing (Pyromark Q24, Qiagen) using variant-specific primers was employed to determine variant heteroplasmy (test sensitivity >3% mutated mtDNA; test specificity ± 2% mutated mtDNA) in tissues.[9]

Results

Genetic investigations of hyperlipidemia

A microarray CGH and trio exome sequencing did not detect a genetic cause.

Developmental milestones of children born after mitochondrial donation

<18 months – normal age appropriate gross and fine motor skills, receptive and expressive language development and social and emotional development in all six children as assessed by parents and interaction with multiple health professionals through routine child health surveillance.

>18 months – Bayley-III scale of infant and toddler development, which encompasses cognitive, gross and fine motor, receptive and expressive language and social/emotional development. Assessment performed on two children who were both found to be within the normal range of expected development in all domains.

>4 years – parental report of normal gross and fine motor skills, normal expressive and receptive language, normal social and emotional development and primary school report of normal academic progress. Assessment of one child.

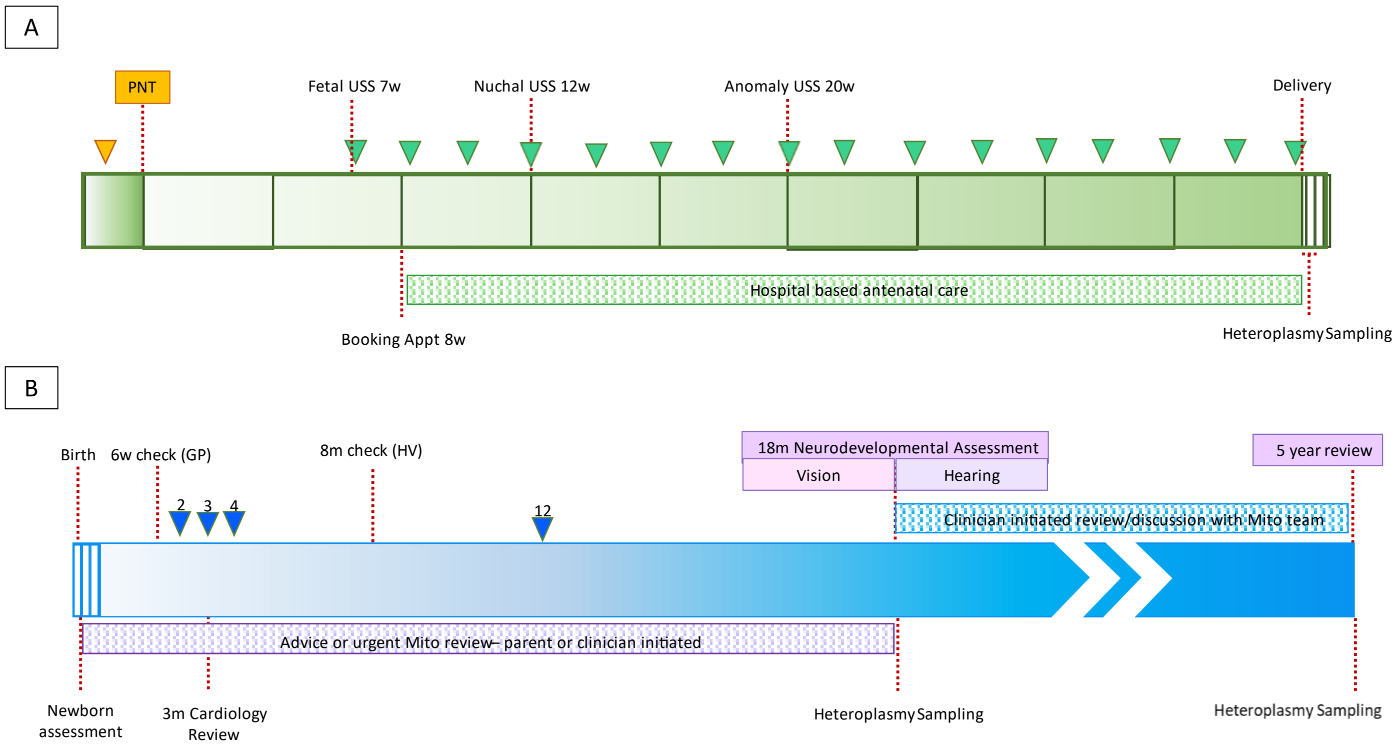

Figure S1

A. **Pregnancy pathway for patients undergoing mitochondrial donation**. The figure presents the timeline for patients starting with consent to follow up (yellow arrow) and then the 9 months of pregnancy. Following confirmation of pregnancy at 7 weeks, the patient has hospital based obstetric care with ultrasound scans (USS) at 12 and 20 weeks. The patient is contacted by the clinical team at 2 weekly intervals (at a minimum) as indicated by green triangles.

B. **Pathway for follow up of child born after mitochondrial donation**. The child is assessed at birth by a paediatrician and then by the local healthcare team at 6 weeks (general practitioner (GP)), at 2, 3 4 and 12 months at time of vaccinations (blue triangles) and at 6 months (health visitor (HV)). At 3 months there is a paediatric cardiology review, and there is access for advice or urgent clinical review by the mitochondrial team. At 18 months there is a formal clinical assessment and measurement of heteroplasmy, followed by another assessment at 5 years just prior to the child starting school.

**Category**	
Disease, problem, or condition under investigation	Mitochondrial DNA disease
Special considerations related to	Mitochondrial DNA disease is transmitted exclusively through the female germline, thus females can pass the disease on to their children but males cannot
Sex and gender	Mitochondrial DNA disease affects both males and females although with some pathogenic variants the disease more frequently manifests in men (for example Leber's Hereditary Optic Neuropathy)
Age	This is a genetic condition that can present at any age
Race or ethnic group	There is no evidence that mitochondrial DNA disease disproportionally affects any particular race or ethnic group.
Geography	Most of the epidemiological and clinical studies have been performed in populations with a European heritage.
Other considerations	Mitochondrial donation is only available to those women who are at risk of transmitting severe mitochondrial DNA disease. In the UK mitochondrial donation treatment requires case-specific approvals from Human Fertilisation and Embryology Authority Statutory Approvals Committee
Overall representativeness of this treatment	The clinical service is available free to all women in the UK with pathogenic mitochondrial DNA variants. The patients described in this study were all female. Those progressing to PGT, or mitochondrial donation had to satisfy specific criteria related to female age, ovarian reserve and health consequences of treatment, pregnancy and childbirth<br><br>The ethnicity of referred women was 90% White, 3.2% Asian, 1% Black, 2.1% Mixed and 3.2% undetermined. For those referred for mitochondrial donation, 94% White and 6% Asian, and for PGT 94% White and 6% Asian. For reference, based on the 2021 census in England and Wales: 82% were White, 9% Asian, 4% Black, 3% Mixed and 2% Others. For our study information on race and ethnicity was available through the medical records

Table S1
Background information on the race, ethnicity, age, and gender of the broader population affected by mitochondrial disease and the relevance of our study

References

1. Hui L, Hayman P, Buckland A, et al. Pregnancy in women with mitochondrial disease-A literature review and suggested guidance for preconception and pregnancy care. Aust N Z J Obstet Gynaecol. 2024. Epub ahead of print.16.

2. Otten ABC, Sallevelt SCEH, Carling PJ, et al. Mutation-specific effects in germline transmission of pathogenic mtDNA variants. Hum Reprod. 2018;33:1331-1341.

3. Greenfield A, Braude P, Flinter F, Lovell-Badge R, Ogilvie C, Perry ACF. Assisted reproductive technologies to prevent human mitochondrial disease transmission. Nat Biotech 2017;35:1059-1068.

4. Kang, Wu J, Gutierrez NM, et al. Mitochondrial replacement in human oocytes carrying pathogenic mitochondrial DNA mutations. Nature 2016;540:270–275

5. Costa-Borges N, Nikitos E, Späth K, et al. First pilot study of maternal spindle transfer for the treatment of repeated in vitro fertilization failures in couples with idiopathic infertility. Fertil Steril 2023;119:964-973

6. Wang, P., Zhao, C., Xu, W. et al. The association between the number of oocytes retrieved and cumulative live birth rate in different female age strata. Sci Rep 2023;13:14516

7. Frederiksen LE, Ølgaard SM, Roos L, et al. Maternal age and the risk of fetal aneuploidy: A nationwide cohort study of more than 500 000 singleton pregnancies in Denmark from 2008 to 2017. Acta Obstet Gynecol Scand. 2024;103:351-359.

8. https://portal.hfea.gov.uk/media/yrkn55xa/2024-10-01-hfea-code-of-practice-v9-4.pdf

9. Clinical Indication R300.1; https://www.england.nhs.uk/publication/national-genomic-test-directories/

10. https://www.acgs.uk.com/media/12533/uk-practice-guidelines-for-variant-classification-v12-2024.pdf

11. Mavraki E, Labrum R, Sergeant K, et al Genetic testing for mitochondrial disease: the United Kingdom best practice guidelines. Eur J Hum Genet 2023;31:148-163.

12. Bayley N. Bayley Scales of Infant and Toddler Development, Third Edition (Bayley-III). Third edition technical manual. Pearson PsychCorp; 2006a.
